# Rapid Integration of Multi-copy Transgenes Using Optogenetic Mutagenesis in *Caenorhabditis elegans*

**DOI:** 10.1534/g3.118.200158

**Published:** 2018-04-24

**Authors:** Kentaro Noma, Yishi Jin

**Affiliations:** * Division of Biological Sciences, Neurobiology Section, University of California, San Diego, La Jolla, California 92093; † Howard Hughes Medical Institute, University of California, San Diego, La Jolla, CA 92093

**Keywords:** *C. elegans*, transgene integration, optogenetic mutagenesis, miniSOG

## Abstract

Stably transmitted transgenes are indispensable for labeling cellular components and manipulating cellular functions. In *Caenorhabditis elegans*, transgenes are generally generated as inheritable multi-copy extrachromosomal arrays, which can be stabilized in the genome through a mutagenesis-mediated integration process. Standard methods to integrate extrachromosomal arrays primarily use protocols involving ultraviolet light plus trimethylpsoralen or gamma- or X-ray irradiation, which are laborious and time-consuming. Here, we describe a one-step integration method, following germline-mutagenesis induced by mini Singlet Oxygen Generator (miniSOG). Upon blue light treatment, miniSOG tagged to histone (Histone-miniSOG) generates reactive oxygen species (ROS) and induces heritable mutations, including DNA double-stranded breaks. We demonstrate that we can bypass the need to first establish extrachromosomal transgenic lines by coupling microinjection of desired plasmids with blue light illumination on Histone-miniSOG worms to obtain integrants in the F_3_ progeny. We consistently obtained more than one integrant from 12 injected animals in two weeks. This optogenetic approach significantly reduces the amount of time and labor for transgene integration. Moreover, it enables to generate stably expressed transgenes that cause toxicity in animal growth.

Transgenesis provides a powerful means to transfer desired genetic materials into an organism. Transgenes expressing fluorescent reporters are essential for visualization of tissues, cells, subcellular structures or protein localizations; and transgenes overexpressing a gene of interest are necessary to investigate genetic pathways in many biological processes. In *Caenorhabditis* *elegans*, transgenesis can be quickly accomplished following microinjection of DNAs into the germline, which then form heritable extrachromosomal arrays (Mello *et al.* 1991; Evans 2006). Such extrachromosomal transgenes, however, frequently display mosaic expression due to stochastic loss during cell division in somatic and germline cells, as well as variability in expression levels. While Clustered Regularly Interspersed Short Palindromic Repeats (CRISPR)-based knock-in (Kim *et al.* 2014; Paix *et al.* 2014; Paix *et al.* 2015) and Mos1-mediated Single-Copy Insertion (MosSCI) (Frøkjær-Jensen *et al.* 2008; Frøkjær-Jensen *et al.* 2014) have tremendous value for stable expression from endogenous or defined loci, expression levels of such transgenes might be too low for visual detection or may not induce desired overexpression. Thus, integration of high-copy extrachromosomal transgenes remains necessary to achieve stable and high expression. Traditionally, ultraviolet with trimethylpsoralen (UV/TMP) or gamma- or X-ray irradiation has been used to integrate transgenes (Evans 2006). These methods are laborious and time-consuming, requiring the establishment of extrachromosomal transgenic lines before integration (Figure 1A).

**Figure 1:**
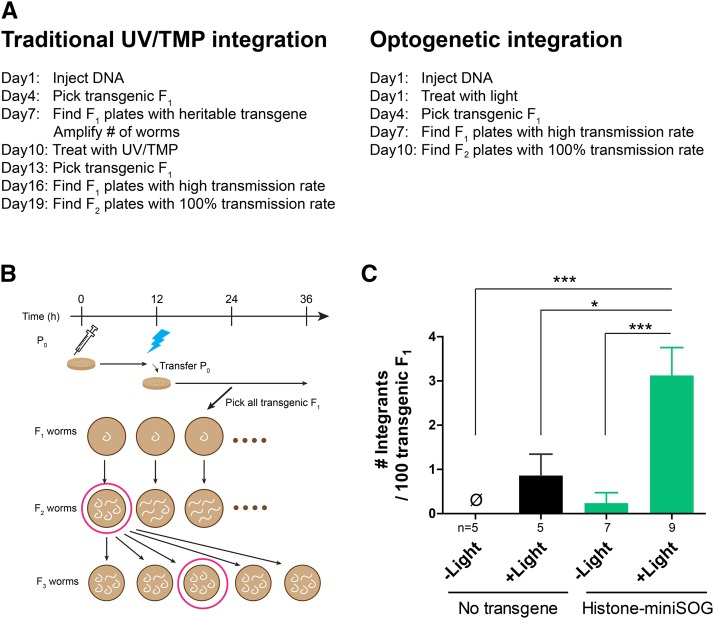
Schematic and efficiency of optogenetic integration. (A) Comparison of the workflow between a traditional UV/TMP method and optogenetic integration. (B) Schematic of optogenetic integration with roller worms as an example of transgenic worms. Illuminating worms with blue light 12 hr after microinjection, parental worms were transferred to new plates. A few days later, transgenic F_1_ worms were singly plated. From F_1_ plates with high transmission rate, five transgenic F_2_ worms were singly plated and examined for 100% transmission rate. The progeny before light treatment was also examined to determine the light dependency of the method. (C) Efficiency of integration was calculated as the number of integrants per 100 transgenic F_1_ progeny. n indicates the number of experiments (12 worms for microinjection/experiment). Error bars indicate S.E.M. Statistics: one-way ANOVA followed by Dunnett’s test, *P < 0.05, ***P < 0.001.

The Light-oxygen-voltage-sensing (LOV) domain-derived Mini Singlet Oxygen Generator (miniSOG) generates reactive oxygen species (ROS) upon blue-light illumination (Shu *et al.* 2011). Previously, we have established a method called “optogenetic mutagenesis”, in which miniSOG fused to a histone is expressed in the germline of *C. elegans*, and induces heritable mutations following blue-light illumination with the estimated loss-of-function mutation frequency to be 0.72 ± 0.14 per 1,000 haploid genomes (Noma and Jin 2015; Noma and Jin 2016). We showed that this optogenetic mutagenesis could also induce integration of extrachromosomal transgenes at a frequency comparable to that using the traditional UV/TMP protocol (Noma and Jin 2015). Here, we significantly improved this approach and demonstrated that establishment of extrachromosomal transgenic lines can be bypassed by coupling microinjection of DNA plasmids with optogenetic mutagenesis in the parental generation. On average, we obtained more than one integrant from the progeny of twelve injected parental worms in two weeks. Moreover, by circumventing the establishment of extrachromosomal transgenic lines, we show that this method has the benefit to integrate transgenes expressing proteins toxic to the development and growth of the organism.

## Materials and Methods

### Strain maintenance

*Caenorhabditis elegans* strains were maintained at 22.5° on Nematode Growth Medium (NGM) plates with OP50 bacteria as previously reported (Brenner 1974). Worms were maintained in the dark except picking for maintenance and imaging. Although the Histone-miniSOG strain does not cause detectable amount of mutations with ambient light (Noma and Jin 2015), it is advised to remove the Histone-miniSOG transgene as well as other background mutations by outcrossing the integrants.

### Plasmids and strains

Bristol N2 was used as the wild type strain. For microinjection and integration, we used CZ20310 *juSi164[Pmex-5-his-72*::*miniSOG-3′ UTR(tbb-2) + C. briggsae* (Cbr)-unc-119(+)*]* *III unc-119(ed3) III *(Noma and Jin 2015), which expresses the single-copy Histone-miniSOG transgene under the control of germline-active promoter and 3′UTR. In case a desired genetic background should be introduced on the chromosome III, we also generated a strain, NUJ4 *knjSi1[Pmex-5-his-72*::*miniSOG-3′UTR(tbb-2)] IV*, which carries the single-copy Histone-miniSOG transgene. *rol-6(su1006dm)* (pRF4) (Mello *et al.* 1991) and P*ttx-3*-RFP-3′UTR(*unc-54*) plasmids were used to examine the efficiency of optogenetic integration. For GFP::RPS-18 expression, P*rps-18*-GFP::*rps-18* plasmid (pCZGY3162) (Noma *et al.* 2017) was used. To enhance the GFP::RPS-18 signals (Noma *et al.* 2017), *juIs531[GFP*::*rps-18 + rol-6(su1006dm)]* transgene was examined in the *rps-18(ok3353)/nT1[qIs51 (Pmyo-2*::*GFP + Ppes-10*::*GFP + PF22B7.9*::*GFP)]* background. P*rgef-1*-GFP::*efa-6*N-3′UTR(*unc-54*) (pLC665) was made by recombination between GFP::*efa-6*N entry vector and P*rgef-1*-GTW destination vector using Gateway system (Invitrogen). pLC665 contains exons corresponding to the first 150 amino acids of EFA-6, which was fused to GFP with a 22 amino acid linker. For overexpressing *dlk-1*, we used pCZ848 expressing P*rgef-1*-GFP::DLK-1L and *Cbr-unc-119*, which was generated for MosSCI insertion (Holland *et al.* 2016). We note that *Cbr-unc-119* overexpression does not cause uncoordinated behavior.

### Microinjection and optogenetic integration

Plasmid DNAs for microinjection were purified with QIAprep Spin Miniprep Kit (Qiagen), mixed at 100 ng/µl in total unless otherwise noted, and centrifuged at 15,000 rpm for 10 min at room temperature to avoid clogging microinjection needles. The plasmid solution was injected into the gonad of CZ20310 as previously described (Mello *et al.* 1991). We used non-gravid young adults for microinjection, which could be obtained by culturing worms at 22.5° approximately 50 hr post egg laying. Older worms can be used but the brood size would be decreased. Six to eighteen hours after microinjection, we illuminated worms with blue light at 2 mW/mm^2^ and 4 Hz for 30 min as previously described (Noma and Jin 2015; Noma and Jin 2016). These P_0_ worms were recovered on new seeded plates after light illumination. A few days later, all transgenic F_1_ worms were put on individual plates (F_1_ plates). Among these F_1_ plates, we selected the plates with >50% F_2_ worms carrying transgenes and recovered single transgenic F_2_ onto five individual plates. A few days later, we examined for 100% transgene-positive F_3_ worms. The potential integrated strains were crossed to N2 males and confirmed for Mendelian segregation. We did not find any integrants having two integration sites based on the segregation. We note that integrants may not show 100% penetrance of phenotypes as described in Results and Discussion. To distinguish between true integrants and extrachromosomal array lines with high-transmission rate, we used a few ways in addition to outcross. First, if the transgenes have fluorescence, we examined them under an epifluorescence upright microscope instead of a fluorescent stereo scope. Integrants sometimes show 100% fluorescence of the transgene despite variable expression of a co-injection marker. Second, we picked animals without phenotypes of the co-injection marker and checked if they produced transgenic progeny. True integrants will produce marker-positive progeny from marker-negative parents.

### Quantitative PCR

Twenty adults were picked and put into the lysis solution (10 mM Tris (pH 8.8), 50 mM KCl, 0.1% Triton X-100, 2.5 mM MgCl_2_, 100 µg/mL Proteinase K). These worms were then lysed by incubating at 65° for 60 min and subsequently at 95° for 15 min for inactivation of Proteinase K. Two microliters of the worm lysis were used as the templates for quantitative PCR (qPCR) using iQ SYBR Green Supermix (Bio-Rad) and a C1000 thermal cycler (Bio-Rad) with CFX96 Real-Time system (Bio-Rad). The parameters for the qPCR were 95° for 3 min and 40 cycles of 95° for 10 sec and 55° for 30 sec. The following primers were used for detecting integrated transgenes as well as *rps-25* for the internal control: *rps-25*:

YJ12209 (TCACACCATCCGTCGTCTCTG) and YJ12211 (GACCTGTCCGTGATGATGAACG), *ttx-3*: YJ12212 (GAGCATACGCTTCGTCGGACT) and YJ12213 (GGAAAGTCATGTTGCGCGAGAG), *rol-6*: YJ12214 (AGGAAGGACCAGATGGGCAC) and YJ12215 (GGTGAAGCATCCTGTTGGTGG), *efa-6*: YJ12216 (GCATCGAGCATTCACCCACATC) and YJ12217 (CGATGGAAACGAAACTCCCGC). Copy number of integrated transgenes was calculated by normalizing to N2 and *rps-25*.

### Microscopy

Bright field images and a movie were obtained from young adults using a Stemi508 stereo microscope (Zeiss) and a digital camera L-835 (Hozan). For fluorescence imaging, worms were immobilized with 25%(v/v) of 50 nm polystyrene beads (Polysciences, Inc.) in M9 solution. Representative images were collected from young adults using a LSM710 confocal microscope (Zeiss) equipped with a 10x (NA = 0.3), 63x (NA = 1.4), or 100x (NA = 1.46) objective lens. The zoom function of ZEN software (Zeiss) was used for imaging cell bodies of mechanosensory ALM neurons. Single-plane images or maximum-intensity-projections obtained from several z-sections (0.5 or 1 µm/section) using ZEN software (Zeiss) were shown. Fluorescence intensities were analyzed after subtracting background signals using FIJI software (Schindelin *et al.* 2012).

### Statistical analysis

We used one-way ANOVA followed by Dunnett’s test to compare multiple samples and linear regression to examine correlation in GraphPad Prism 7.0 (GraphPad Software, La Jolla, CA).

### Data availability

The authors state that all data necessary for confirming the conclusions presented in the article are represented fully within the article. Supplemental material available at Figshare: https://doi.org/10.25387/g3.6127709.

## Results and Discussion

### Transgene integration can be coupled with plasmid microinjection

To bypass the establishment of transgenic lines carrying extrachromosomal arrays, we tested if coupling microinjection into the germline with miniSOG-mediated optogenetic mutagenesis could lead to integration of transgenes. We performed microinjection of plasmid DNAs containing *rol-6(su1006dm)*, which produces dominant roller (Rol) phenotype (Mello *et al.* 1991), and those containing P*ttx-3*-RFP, which labels AIY neurons, into the gonad of non-gravid adult hermaphrodites expressing single-copy Histone-miniSOG transgene in the germline (P*mex-5-his-72*::miniSOG, see Materials and Methods)(Noma and Jin 2015). Microinjected P_0_ animals were recovered on seeded for 6, 12, or 18 hr. We then illuminated these P_0_ animals with blue light to activate miniSOG. Nuclear ROS induction by Histone-miniSOG probably causes double-strand breaks, which enable integration of microinjected plasmids (Noma and Jin 2015). Rol F_1_ progeny from light-treated P_0_ was singly propagated (F_1_ plates), and the ratio of Rol F_2_ progeny was examined (Figures 1A and 1B). In theory, if an F_1_ animal has a heterozygous integrated transgene (Rol/+), ∼75% of its F_2_ worms should show Rol phenotypes. To avoid overlooking, we propagated any transgene-bearing F_2_ worms with >50% transmission rate (Table 1, High F_2_). If singly propagated Rol F_2_ worms (F_2_ plates) produced 100% Rol F_3_ progeny, we treated them as potential integrants and proceeded to outcrossing of the F_3_ lines to wild type, followed with confirmation of the Mendelian segregation of Rol. In all integrants identified by Rol, we observed red fluorescence in AIY neurons. These results suggest that the *rol-6(su1006dm)* and P*ttx-3*-RFP transgenes were co-integrated in the genome. We were able to obtain such integrants among progeny of P_0_ animals that received blue light treatment 6, 12, or 18 hr after microinjection (Table 1). The highest number of integrants was obtained from P_0_ treated 6 hr after microinjection, while a slightly higher ratio of integrants per transgenic F1 was obtained from P_0_ treated 12 hr after microinjection. As a control for blue-light dependency, we examined the F_1_ progeny produced from the same P_0_ animals before light treatment (Figure 1B and Table 1, no light), and found only one integrant from a total of 227 transgenic F_1_ animals, which was likely generated spontaneously. In contrast, we obtained ten integrants from a total of 410 transgenic F_1_ animals in the light-treated condition (Table 1, light), suggesting that the integration is light-dependent (see below). We named this process as “optogenetic integration”.

**Table 1:** Optogenetic integration with light treatment at different time after injection

		No light, # transgenic animals				Light, # transgenic animals			
Time (h)*^a^*	# P_0_*^b^*	F_1_	F_2_	High F_2_	Integrant	F_1_	F_2_	High F_2_	Integrant
6	12	0	0	0	0	114	45	31	1
6	12	2	0	0	0	143	40	13	5
12	12	33	7	4	0	34	14	10	2
12	12	60	32	20	1	74	34	22	1
18	12	78	41	29	0	25	8	7	0
18	12	54	25	14	0	20	5	5	1
	Total:	227	105	67	1	410	146	88	10

a Time (h) = time of light illumination after injection.

b # P_0_ = number of injected animals.

The total plasmid concentration is 100 ng/µm for all conditions.

### Optogenetic integration is efficient

We further quantified the efficiency of optogenetic integration. From 12 P_0_ worms treated with blue light 12 hr after microinjection, we obtained 61.3 ± 11.5 transgenic F_1_ worms (mean ± SEM, n = 9 experiments), and 12.2 ± 2.5 F_2_ animals carrying germline transmittable transgenes with >50% transmission rate. Among them, we obtained 1.6 ± 0.3 integrants. Statistical analysis showed that the optogenetic integration depends on both Histone-miniSOG transgene and light illumination (Figure 1C). The efficiency calculated as the number of integrants per 100 F_1_ transgenic worms (Figure 1C) was approximately five times higher than that using UV/TMP method or using previously reported miniSOG-based method (Noma and Jin 2015).

We next examined the effect of the total plasmid DNA concentration for microinjection on the efficiency. Extrachromosomal arrays are not efficiently transmittable to F_2_ generation when the total concentration of plasmids is lower than 100 ng/µl (Mello *et al.* 1991). Here, we reasoned that by microinjecting lower concentration of plasmids, the integrants might be enriched among transgenic F_2_. To test this, we microinjected the plasmids with the total concentration of 10 ng/µl or 25 ng/µl to CZ20310 worms and illuminated them with blue light 12 hr after microinjection. As expected, the efficiency to obtain heritable F_2_ transgenic animals was reduced (Table 1 and Table S1). However, the efficiency to obtain integrants was also significantly reduced, compared to the microinjection with 100 ng/µl of total plasmids (Table 1 and Table S1). Therefore, we conclude that about 100 ng/µl of plasmids in total is favorable for optogenetic integration.

### Optogenetic integration produces high-copy transgenes

**Figure 2:**
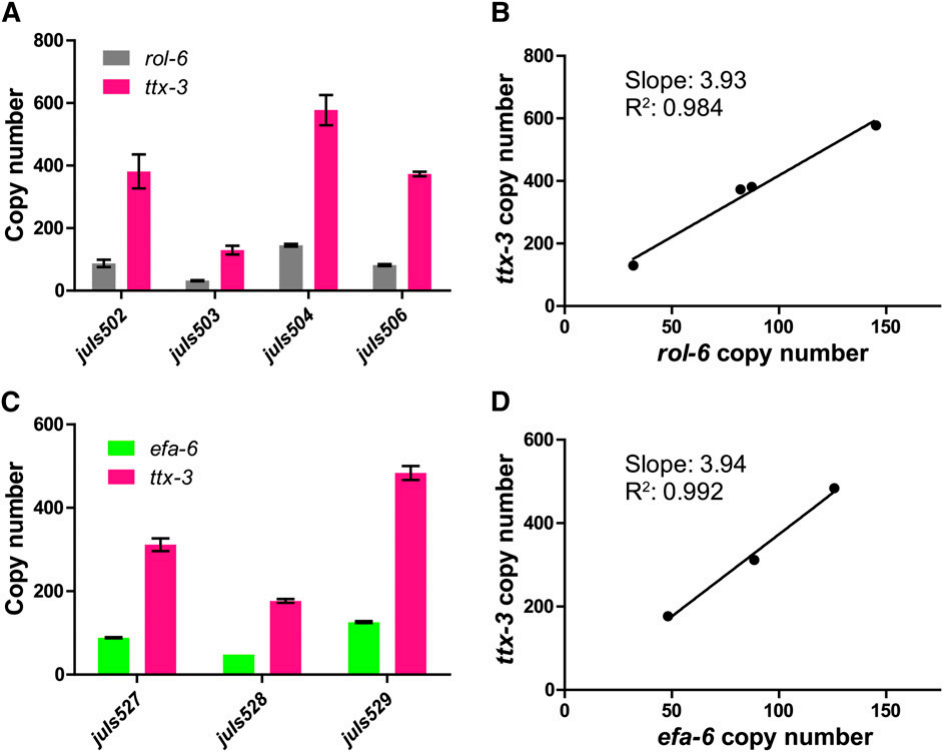
Copy number of optogenetically integrated transgenes. *rol-6(su1006dm)* at 25 ng/µl for (A) and (B) or P*rgef-1*-GFP::*efa-6*N at 25 ng/µl for (C) and (D) were microinjected with P*ttx-3*-RFP at 75 ng/µl. (A and C) The copy number was examined using quantitative PCR. Error bars indicate S.E.M. N = 2 biological replicates. (B and D) The ratio between two plasmids is consistent among different transgenes.

We determined the copy number of the transgenes using quantitative PCR (qPCR). Extrachromosomal arrays are estimated to contain hundreds of copies of injected plasmids (Stinchcomb *et al.* 1985; Mello *et al.* 1991). We found that the copy number of optogenetically integrated transgenes was roughly correlated with the concentration of microinjected plasmids; the copy number of P*ttx-3*-RFP per haploid genome ranged from 129 to 576 when injected at 75 ng/µl, while those of *rol-6(su1006dm)* ranged from 31 to 144 when injected at 25 ng/µl (Figure 2A). In contrast to the variation of the copy number of each plasmid, the ratio between two plasmids from the same microinjection condition was consistent among independently isolated integrants (Figure 2B). This trend was also observed in another combination of plasmids (P*ttx-3*-RFP and P*rgef-1*-GFP::*efa-6*N, Figures 2C and 2D, see below). Thus, the optogenetic approach is suitable for integrating high-copy transgenic arrays and the desired copy number can be achieved by varying concentration of DNA plasmids. The high copy number and constant ratio of two co-injected plasmids suggest that a mixture of microinjected plasmids was integrated as a single transgenic array. Formation of extrachromosomal arrays appear to occur rapidly, within less than six hours, because we obtained integrants by light treatment six hours after microinjection.

### Optogenetically integrated transgenes are stably expressed

**Figure 3:**
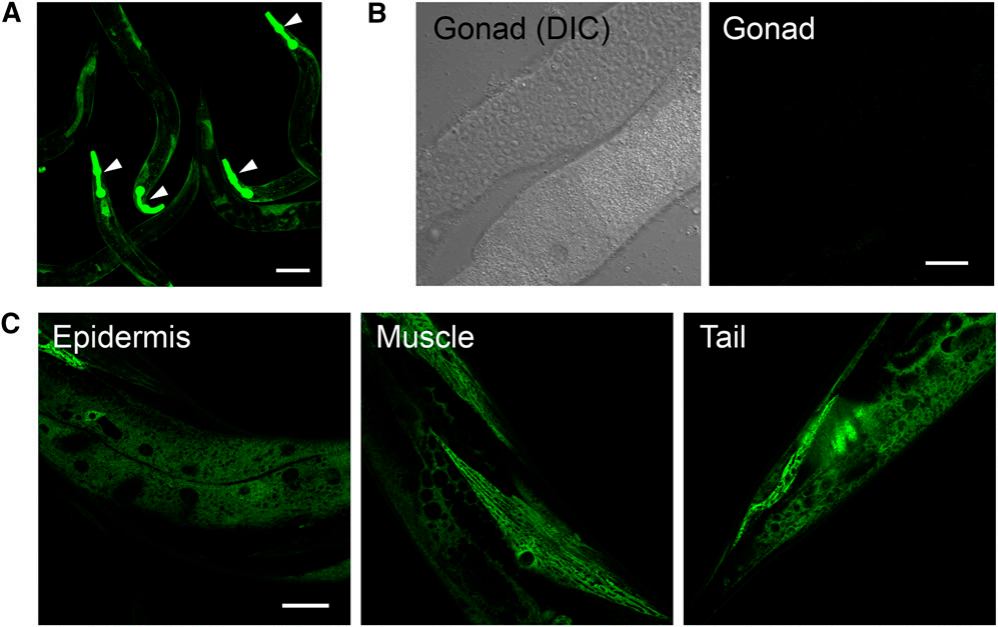
Optogenetically integrated transgenes are stably expressed. Expression of *juIs531[GFP*::*RPS-18]*, which has one copy of integrated transgene in the background of *rps-18(ok3353)/nT1[qIs51 (Pmyo-2*::*GFP + Ppes-10*::*GFP + PF22B7.9*::*GFP)]*. (A) Live animals showing the stable expression among individuals. White arrowheads indicate the pharyngeal GFP signals expressed from nT1 balancer. (B) Dissected gonad showing the lack of expression. (C) Live animals showing the expression in the somatic tissues. Scale bars: 100 µm in (A), 20 µm in (B) and (C).

We examined if optogenetically integrated transgenes showed consistency in expression level and pattern using a ubiquitously expressed ribosomal small subunit protein 18 (*rps-18*) (Noma *et al.* 2017). We generated two integrants expressing GFP-tagged *rps-18* with a co-injection marker *rol-6(su1006dm)* (*juIs531* and *juIs532[Prps-18-GFP*::*rps-18 + rol-6(su1006dm)]*). We detected consistent GFP::RPS-18 expression among different individuals from a single parent (Figure 3A) in all somatic tissues (Figure 3C). We note, however, that other integrated transgenes, such as *juIs504 and juIs505[rol-6(su1006dm) + Pttx-3-RFP]*, showed stochastic expression of RFP in AIY, despite that Rol phenotype was 100% penetrant (Figure S1). This stochastic expression is probably not due to optogenetic integration protocol because variable expression is also reported in the integrants generated by traditional methods (Mello and Fire 1995). Since the insertion site in the genome is random in optogenetic integration, it is possible that integrated transgenes are silenced due to the position effect on the chromosome (Hsieh and Fire 2000). CRISPR-based insertion method might be used to incorporate multi-copy transgenes into a defined locus (Yoshina *et al.* 2016).

In *C. elegans* high-copy transgenes are often silenced in the germline cells (Kelly *et al.* 1997). We previously showed that a single copy GFP::*rps-18* transgene made by MosSCI technique is expressed in the germline (Noma *et al.* 2017). Here, we did not observe expression of GFP::RPS-18 in the germline from two transgenes *juIs531* and *juIs532* that were generated by microinjecting P*rps-18*-GFP::*rps-18* plasmids at 1 ng/µl and had only one and six copies of GFP::*rps-18* per haploid genome, respectively. For reliable germline expression, optogenetic integration method might be combined with complex arrays (Kelly *et al.* 1997) or introns with Periodic An/Tn-Clusters (PATCs) (Frøkjær-Jensen *et al.* 2016).

### Optogenetic integration of transgenes toxic to organism development and/or function

**Figure 4:**
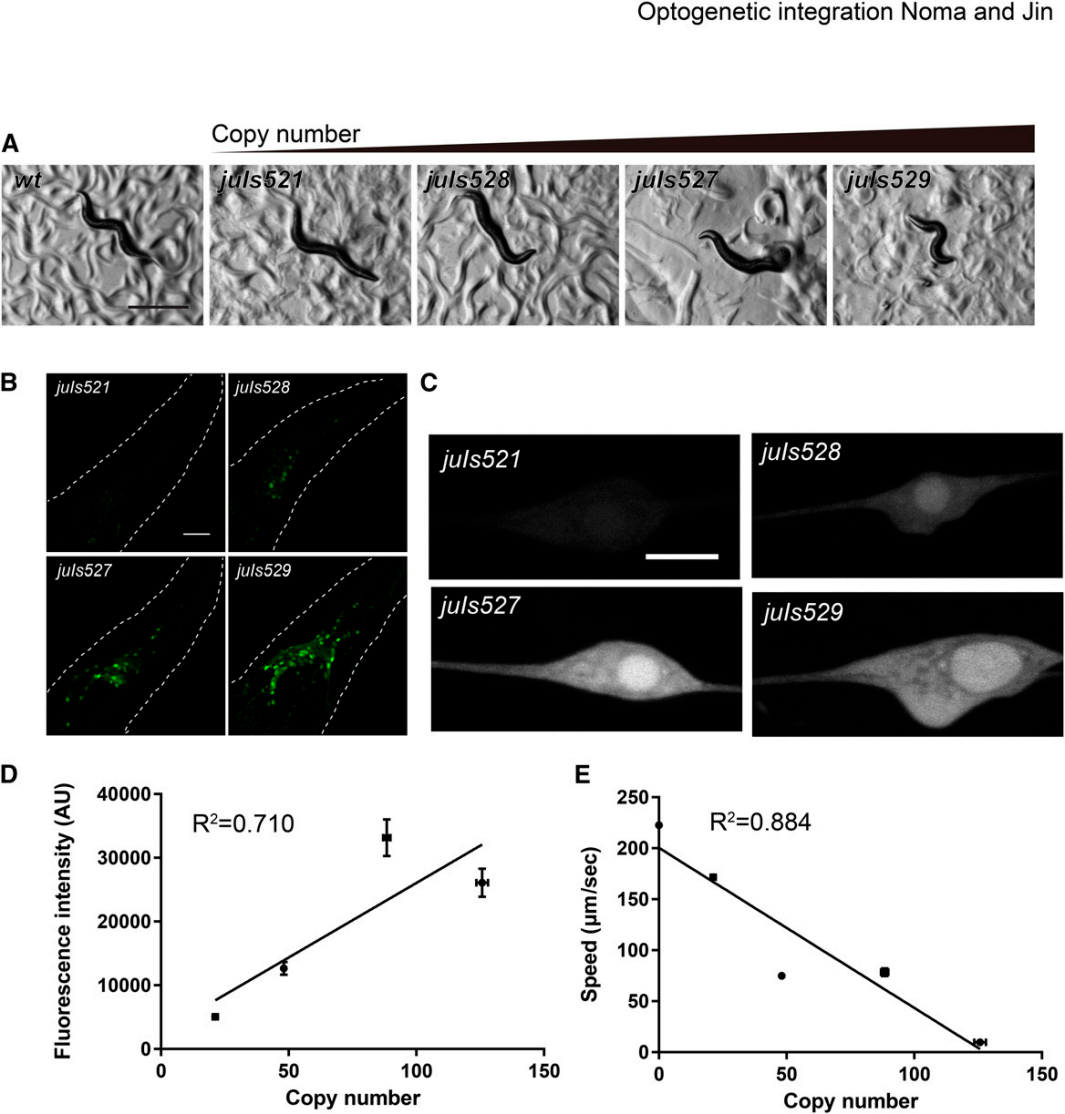
EFA-6N overexpression makes worm uncoordinated in a dose dependent manner. P*rgef-1*-GFP::EFA-6-N-terminus (GFP::EFA-6N) was overexpressed from the optogenetically integrated transgenes. (A) Bright field images of adult worms showing that overexpression of EFA-6N caused uncoordination depending on the copy number of integrated GFP::EFA-6N. Scale bar: 500 µm. (B and C) Fluorescent images of the heads (B) and ALM neurons (C) showing different expression levels of GFP::EFA-6N. Dotted lines in (B) indicate the outlines of heads. Scale bars: 20 µm in (B) and 5 µm in (C). (D and E) Copy number of integrated transgenes was plotted against the fluorescence intensity in ALM neurons (D), and against the speed of worm locomotion (E). Error bars indicate S.E.M.

Traditional methods for transgene integration require establishment of transgenic lines before mutagenesis, which is not suitable for integrating transgenes that cause sickness of worms. For example, overexpression of a dominant-interfering N-terminus of Exchange Factor for ARF-6 (EFA-6N) in neurons inhibits axon regeneration and perturbs neuronal development (Chen *et al.* 2011; Chen *et al.* 2015). While we could obtain extrachromosomal transgenes expressing the EFA-6N in all neurons, these worms had small brood size and displayed uncoordinated movement. In repeated efforts following UV/TMP method, we failed to obtain any integrants. We reasoned that optogenetic integration might overcome this problem because it bypasses the establishment of extrachromosomal transgenic lines. Indeed, from 24 injected P_0_ using optogenetic integration, we successfully obtained four integrants of P*rgef-1*-GFP::*efa-6*N, which expresses GFP::EFA-6N under the control of pan-neuronal *rgef-1* promoter (Altun-Gultekin *et al.* 2001). Interestingly, different GFP::EFA-6N integrants had different degree of uncoordination (Figure 4A). We examined the relationship between the copy number and the fluorescence intensity or the uncoordinated behavior (Figure 4). The copy number was positively correlated with the fluorescence intensity (Figures 4B-D) and negatively with the speed of the worms (Figure 4E). Thus, the variation in copy number of optogenetic integration enables to address the dose-dependency of transgene expression. Similar to EFA-6N, overexpression of the active long isoform of dual-leucine zipper kinase (DLK-1L) in all neurons causes severe uncoordinated behavior (Nakata *et al.* 2005; Yan and Jin 2012), making it difficult to integrate extrachromosomal arrays with high expression level. Using optogenetic integration, we obtained one integrant expressing DLK-1L in all neurons (*knjIs1[Prgef-1-GFP*::*DLK-1L +* *Cbr-unc-119* *+ Pttx-3-RFP])* from 12 P_0_ animals, and these transgenic animals showed uncoordinated behavior (Figure S2 and Movie S1). Thus, optogenetic integration is suitable for the integration of transgenes with toxicity to organism development and/or function.

In summary, we have established a one-step optogenetic integration method that bypasses the need to first establish transgenic extrachromosomal arrays lines. This technical improvement has two benefits. One is significant reduction of time and labor for integration. Research using *C. elegans* has a strong demand on fast and efficient integration for rapid expansion of newly available reporters and genetic tools. For example, cGAL4-UAS system has recently become available in *C. elegans* community (Wang *et al.* 2017). To fully utilize this system, it is necessary to generate many different effector and promoter-driven GAL4 strains with high and stable expression. The second benefit is that optogenetic integration makes it feasible to obtain integrants of high-copy transgenes that have toxic effects to organisms. Such transgenes are often desired in forward genetic screens to find the genetic modifiers of target genes (Wang and Sherwood 2011). For example, the generation of transgenes that fully mimic toxic effects of disease-causing mutations is useful for research using *C. elegans* as models for neurodegenerative diseases (Teschendorf and Link 2009; Markaki and Tavernarakis 2010).

## References

[bib1] Altun-Gultekin Z. Andachi Y. Tsalik E. L. Pilgrim D. Kohara Y., 2001 A regulatory cascade of three homeobox genes, *ceh-10, ttx-3,* and *ceh-23*, controls cell fate specification of a defined interneuron class in *C. elegans*. Development 128: 1951–1969. 11493519 10.1242/dev.128.11.1951

[bib2] Brenner S., 1974 The genetics of *Caenorhabditis elegans*. Genetics 77: 71–94. 4366476 10.1093/genetics/77.1.71 PMC1213120

[bib3] Chen L. Chuang M. Koorman T. Boxem M. Jin Y., 2015 Axon injury triggers EFA-6 mediated destabilization of axonal microtubules via TACC and doublecortin like kinase. eLife 4 10.7554/eLife.08695 PMC4596636 26339988

[bib4] Chen L. Wang Z. Ghosh-Roy A. Hubert T. Yan D., 2011 Axon regeneration pathways identified by systematic genetic screening in *C. elegans*. Neuron 71: 1043–1057. 10.1016/j.neuron.2011.07.009 21943602 PMC3183436

[bib5] Evans T. C., 2006 Transformation and microinjection. WormBook ed. The C. elegans Research Community, WormBook. 10.1895/wormbook.1.108.1

[bib6] Frøkjær-Jensen C. Davis M. W. Hopkins C. E. Newman B. J. Thummel J. M., 2008 Single-copy insertion of transgenes in *Caenorhabditis elegans*. Nat. Genet. 40: 1375–1383. 10.1038/ng.248 18953339 PMC2749959

[bib7] Frøkjær-Jensen C. Davis M. W. Sarov M. Taylor J. Flibotte S., 2014 Random and targeted transgene insertion in *Caenorhabditis elegans* using a modified Mos1 transposon. Nat. Methods 11: 529–534. 10.1038/nmeth.2889 24820376 PMC4126194

[bib8] Frøkjær-Jensen C. Jain N. Hansen L. Davis M. W. Li Y., 2016 An Abundant Class of Non-coding DNA Can Prevent Stochastic Gene Silencing in the *C. elegans* Germline. Cell 166: 343–357. 10.1016/j.cell.2016.05.072 27374334 PMC4947018

[bib9] Holland S. M. Collura K. M. Ketschek A. Noma K. Ferguson T. A., 2016 Palmitoylation controls DLK localization, interactions and activity to ensure effective axonal injury signaling. Proc. Natl. Acad. Sci. USA 113: 763–768. 10.1073/pnas.1514123113 26719418 PMC4725513

[bib10] Hsieh J. Fire A., 2000 Recognition and silencing of repeated DNA. Annu. Rev. Genet. 34: 187–204. 10.1146/annurev.genet.34.1.187 11092826

[bib11] Kelly W. G. Xu S. Montgomery M. K. Fire A., 1997 Distinct requirements for somatic and germline expression of a generally expressed *Caernorhabditis elegans* gene. Genetics 146: 227–238. 9136012 10.1093/genetics/146.1.227 PMC1207937

[bib12] Kim H. Ishidate T. Ghanta K. S. Seth M. Conte D. Jr, 2014 A co-CRISPR strategy for efficient genome editing in *Caenorhabditis elegans*. Genetics 197: 1069–1080. 10.1534/genetics.114.166389 24879462 PMC4125384

[bib13] Markaki M. Tavernarakis N., 2010 Modeling human diseases in *Caenorhabditis elegans*. Biotechnol. J. 5: 1261–1276. 10.1002/biot.201000183 21154667

[bib14] Mello C. Fire A., 1995 DNA transformation. Methods Cell Biol. 48: 451–482. 10.1016/S0091-679X(08)61399-0 8531738

[bib15] Mello C. C. Kramer J. M. Stinchcomb D. Ambros V., 1991 Efficient gene transfer in *C. elegans:* extrachromosomal maintenance and integration of transforming sequences. EMBO J. 10: 3959–3970. 1935914 10.1002/j.1460-2075.1991.tb04966.x PMC453137

[bib16] Nakata K. Abrams B. Grill B. Goncharov A. Huang X., 2005 Regulation of a DLK-1 and p38 MAP kinase pathway by the ubiquitin ligase RPM-1 is required for presynaptic development. Cell 120: 407–420. 10.1016/j.cell.2004.12.017 15707898

[bib17] Noma K. Goncharov A. Ellisman M. H. Jin Y., 2017 Microtubule-dependent ribosome localization in *C. elegans* neurons. eLife 6: e26376 10.7554/eLife.26376 28767038 PMC5577916

[bib18] Noma K. Jin Y., 2015 Optogenetic mutagenesis in *Caenorhabditis elegans*. Nat. Commun. 6: 8868 10.1038/ncomms9868 26632265 PMC4686824

[bib19] Noma K. Jin Y., 2016 Optogenetic Random Mutagenesis Using Histone-miniSOG in *C. elegans*. J. Vis. Exp. 117: e54810, 1–7. 10.3791/54810. PMC5226243 27911392

[bib20] Paix A. Folkmann A. Rasoloson D. Seydoux G., 2015 High Efficiency, Homology-Directed Genome Editing in *Caenorhabditis elegans* Using CRISPR-Cas9 Ribonucleoprotein Complexes. Genetics 201: 47–54. 10.1534/genetics.115.179382 26187122 PMC4566275

[bib21] Paix A. Wang Y. Smith H. E. Lee C. Y. Calidas D., 2014 Scalable and versatile genome editing using linear DNAs with microhomology to Cas9 Sites in *Caenorhabditis elegans*. Genetics 198: 1347–1356. 10.1534/genetics.114.170423 25249454 PMC4256755

[bib22] Schindelin J. Arganda-Carreras I. Frise E. Kaynig V. Longair M., 2012 Fiji: an open-source platform for biological-image analysis. Nat. Methods 9: 676–682. 10.1038/nmeth.2019 22743772 PMC3855844

[bib23] Shu X. Lev-Ram V. Deerinck T. J. Qi Y. Ramko E. B., 2011 A genetically encoded tag for correlated light and electron microscopy of intact cells, tissues, and organisms. PLoS Biol. 9: e1001041 10.1371/journal.pbio.1001041 21483721 PMC3071375

[bib24] Stinchcomb D. T. Shaw J. E. Carr S. H. Hirsh D., 1985 Extrachromosomal DNA transformation of *Caenorhabditis elegans*. Mol. Cell. Biol. 5: 3484–3496. 10.1128/MCB.5.12.3484 3837845 PMC369179

[bib25] Teschendorf D. Link C. D., 2009 What have worm models told us about the mechanisms of neuronal dysfunction in human neurodegenerative diseases? Mol. Neurodegener. 4: 38 10.1186/1750-1326-4-38 19785750 PMC2762972

[bib26] Wang H. Liu J. Gharib S. Chai C. M. Schwarz E. M., 2017 cGAL, a temperature-robust GAL4-UAS system for *Caenorhabditis elegans*. Nat. Methods 14: 145–148. 10.1038/nmeth.4109 27992408 PMC5693259

[bib27] Wang Z. Sherwood D. R., 2011 Dissection of genetic pathways in *C. elegans*. Methods Cell Biol. 106: 113–157. 10.1016/B978-0-12-544172-8.00005-0 22118276 PMC4116751

[bib28] Yan D. Jin Y., 2012 Regulation of DLK-1 Kinase Activity by Calcium-Mediated Dissociation from an Inhibitory Isoform. Neuron 76: 534–548. 10.1016/j.neuron.2012.08.043 23141066 PMC3508676

[bib29] Yoshina S. Suhehiro Y. Kage-Nakadai E. Mitani S., 2016 Locus-specific integration of extrachromosomal transgenes in *C. elegans* with the CRISPR/Cas9 system. Biochem. Biophys. Rep. 5: 70–76. 10.1016/j.bbrep.2015.11.017 28955808 PMC5600330

